# State and future of interventional radiology in Malaysia

**DOI:** 10.2349/biij.5.4.e33

**Published:** 2009-10-01

**Authors:** BJJ Abdullah

**Affiliations:** Department of Biomedical Imaging, University of Malaya, Kuala Lumpur, Malaysia

## HEALTHCARE IN MALAYSIA

The fundamental principle of the Malaysian healthcare system is that accessibility to health care should not be related to one’s ability to pay, especially when involving sickness [1]. This is based on the Government’s recognition that health represents human capital, which forms the central thrust to sustainable economic growth and development of the country. The Malaysian healthcare system has been able to achieve a higher standard of health status despite the relatively limited resources available to the health sector (2.0% to 4.0% of GDP). From 1990 to 2005, the life expectancy at birth increased significantly (males from 69.0 years to 71.8 years, females from 73.5 years to 76.2 years) while the infant mortality rate fell (from 13.5 to 5.1 per 1,000 live births) and correspondingly the maternal mortality rate has remained unchanged (at 30 per 100,000 live births) [2]. In the World Health Report 2000 (which assessed the overall health system performance against three objectives of good health, responsiveness and fair financial contribution), Malaysia ranked 49 from 191 WHO member countries [3]. With regards to the Delivery of Health Care, a dual health care system, with both the public and private health services, co-exists in Malaysia. Public health care is provided through government hospitals and health clinics throughout the country. The services range from outpatient curative care to preventive and promotion of health. The main public health provider is Ministry of Health (MOH) that provides primary care, secondary care and tertiary care through various types of health facilities (such as general hospitals, district hospitals and health clinics). In 2008 there were 130 MOH hospitals (with a total of 33,004 beds), 6 special medical institutions (with 5,000 beds), National Institutes of Health (6), 802 health clinics, 1,927 rural clinics, 95 maternal and child health clinics, and 193 mobile clinics [4]. An open-door policy in regard to general outpatient services and hospital admissions has been practiced by the public health sector. Access to specialist services is, nonetheless, controlled through a national system of referral. Specialist services are available at designated hospitals (such as national referral hospital in the capital, the state hospital and selected district hospitals). Referral of patients for specialist services is to the nearest facility if patients cannot be managed at general outpatient facilities. The National Quality Assurance Programme was implemented to maintain, improve and evaluate the quality, efficiency and effectiveness in the delivery of public health services [5]. The Clients Charter commits providers to providing a specified standard of services explicitly and can be used in order to monitor the quality of services and enhance customer satisfaction.

Additional public services are provided by university affiliated and military hospitals. There are seven non-MOH Government Hospitals with 3,245 beds, 209 Private Hospitals with 11,689 beds, and 22,174 and 12,274 Private Maternity and Nursing Homes, respectively. The total number of doctors is 25,102 giving a doctor to population ratio of 1:1,105 but the distribution is skewed towards the urban areas. The Total Expenditure for both the public and private sectors on Health for 2008 was RM30,227,929,810.20 (USD 8,636,551,374.34) which translates to 4.7% of the Total Expenditure on Health as a Percentage of GDP of which approximately 45% was public expenditure. An admission to private hospitals was 1.6 times higher than the public hospitals.

From what was largely a government-led and funded public service enterprise since independence, the Malaysian healthcare service has over the decades (since the 1980s), transformed into a vibrant dual-tiered parallel system, with a sizable and thriving private sector. Thus far we have not approached a unified system with a declared national healthcare policy of offering universal access to every citizen.

The private sector has always attracted both general and family physicians operating individual clinics or by joining more established group practices. The specialists generally join the better-paying more personalised care practices in urban private medical centres. Private healthcare expansion began in earnest during the 1980s, where private hospital beds increased nearly 10-fold (from 1,171 to 10,405 between 1980 and 2003), and the private sector’s share of hospital beds increased from 3.9–5.8% to 23.4–26.7% [6].

## HISTORY OF INTERVENTIONAL RADIOLOGICAL PRACTICE

**Figure 1:**
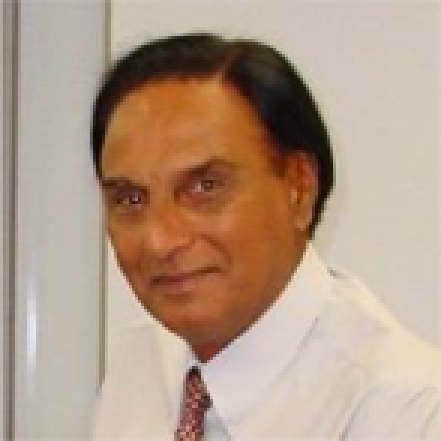
Dr Joginder Singh

Interventional radiology had its origins with the advent of diagnostic angiography, which was first available at University Malaya Medical Centre (formerly University Hospital) in 1968. Four rooms for specialised investigations were established for retrograde pyelography, neuroradiology, angiography and cardiac investigation laboratory. At the time of setting up, there was a critical shortage of radiologists and radiographers in the region, so there was no chance of recruiting trained staff in sufficient numbers to run this large department successfully. The late Professor Emeritus Danaraj initiated an academic staff training scheme, and under this the first radiologist to complete training in London, Dr. Mark Soo, returned to Malaysia in October 1967, followed by others at approximately yearly intervals. The first local Head of Department, Dr. Mark Soo Yoi Sun, was appointed in 1969. The Chair of Radiology was created in 1979 and Dr. Joginder Singh (Figure 1) was appointed the first Professor of Radiology.

**Figure 2:**
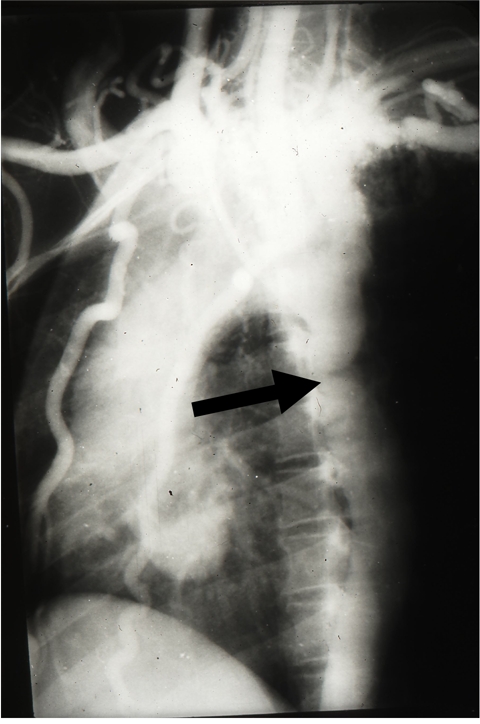
Arch aortogram performed with catheter tip in the aortic arch showing a coarctation (arrows) with the large internal mammary arteries.

One of the early pioneers were Dr. Ah-Hoo Ang and Dr. Joginder Singh who did not have any formal training. The procedures that were being performed initially were pneumoencephalograms, percutaneous transhepatic cholangiograms (PTC), translumbar aortograms and once the Seldinger technique was developed, cardiac and other angiograms (Figure 2). Subtractions had to be done manually always with not so optimal results.

**Figure 3:**
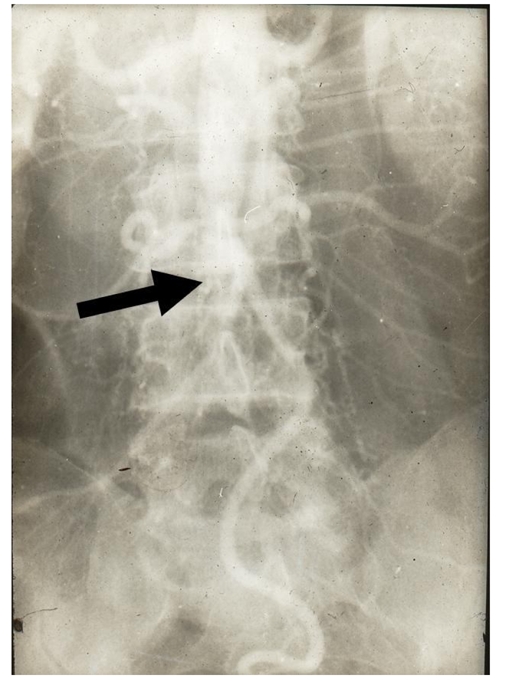
Takayasu’s arteritis showing complete occlusion of the aorta distal to the renals (arrow) with large lumbar and inferior mesenteric artery (IMA) collaterals.

Interestingly, there were a high number of patients with primary arteritis and Bergers’ disease in those days (Figure 3). Carotid angiographies using direct carotid punctures were started in 1971, the same year percutaneous transhepatic biliary drainage (PTBD) procedures were being performed. Nephrostomies were started in 1974/5 when embolisation of lower limb arteriovenous malformations (AVMs) was also being performed using gelfoam. The embolisation provided was only temporary and the patients were given appointments for repeat procedures. Overseas training in Australia taken by Dr. Joginder Singh in 1977, allowed the interventional service to be further expanded.

Pre-shaped catheters were only available in 1978 when recycled catheters were shipped from the USA by some of the interventional radiologists there. Prior to this, the catheters had to be hand-shaped by using steam, which often did not perform the job satisfactorily. Coronary angiographies were started in the early 1980s by Dr. Joginder Singh and Dr. K.T. Singham (a cardiologist who returned from overseas training).

The initial angiography system was donated by the Australian Government under the Colombo plan. This used roll film had to be viewed on a film roller. This was followed by a “puck-changer” angiography system in the late 1970s. With the advent of the CT scanner in 1980, the number of angiograms dropped tremendously. The resources were made available by the medical centre and it was the full complement of other specialists’ i.e. cardiothoracic surgeons, physicians, etc, which made growth possible. Dr. Ang migrated to Australia in 1976 while Dr. Joginder retired in 1995 following which Dr. Abdullah Daud took over.

Dr. Samad Sajikan who was seconded to the Universiti Kebangsaan Malaysia (UKM) from the MOH, was responsible for taking the practice of interventional radiology to the next level from the mid-1990s. Eventually holding the position of Professor and Head of the Radiology Department in UKM, he was instrumental in making interventional radiology and interventional radiologists recognised as a vital component of medical practice not only in the Kuala Lumpur General Hospital (KLGH) and the Universiti Hospital UKM (which were sharing the same premises) but in the entire country. They were performing hepatobiliary, urological and pulmonary interventions. Inferior vena cava (IVC) filters were implanted in 1995. Funding was initially difficult and replacement of the aging equipment was also challenging. There was little neuro and peripheral vascular work at that time.

Dr. Samad was the role model who inspired the younger radiologists with his passion, knowledge, skills and dedication. His network of international interventional radiologists in Japan, UK, USA, etc, was also one of his strengths. Dr. Samad was the nidus around which the next generation of interventional radiologists sprouted and grew. Dr. Samad is currently in private practice and he can be truly called the “father” of interventional radiology in Malaysia. His mantle in UHUKM was then taken over by Dr. Nafikuddin Hj. Mahmud while the KLGH services were taken over by Dr. Alex Tang (who incidentally was probably one of the first formally trained interventional radiologist).

Interventional radiology in the University Hospital of the Science University of Malaysia (UHUSM) was pioneered by Dr. Nurul Azman Ahmad Alias who trained under Dr. Samad in UKM. He returned to Kota Bharu, Kelantan, and was fortunate to have a surgeon who was supportive both in terms of referring patients and also looking after the patients following the interventional procedures. The early days in UHUSM were troubled with old and, frequently, non-functioning equipment. The department then managed to secure funding from the university and they were able to move along. They were fortunate to have supportive interventional radiologists, both locally (e.g. Dr. Adam Pany) and regionally who were prepared to visit them regularly to help them learn the newer techniques. Today, they are busy doing a full range of interventional producers.

## CURRENT STATUS

It has only been in the last decade or so when the number of formally trained interventional radiologists has increased in Malaysia. This has been attributed to an increased awareness of the important role interventional radiology plays in medical practice by the leaders in radiology as well as the support shown by the early leaders in interventional radiology. The training has been performed overseas especially in the UK, Australia and Singapore.

There are practicing interventional radiologists in all three sectors i.e. the public, private as well as the universities. The total number of trained interventional radiologists is currently approximately 20 with the majority within the universities, followed by the private hospital and the fewest within the MOH. There is a constant move of specialists from the MOH to the private sector and this has hampered the development of interventional radiology within the MOH. It is anticipated that this trend will persist. It is anticipated that it will be at least 5 years before any significant changes are seen.

It is heartening to note that over the last decade the number of radiologists has increased significantly and this has enabled most public and private hospitals to build up their numbers and provide a higher level of service. It is believed that as these numbers increase, it will free some of them from the heavy diagnostic imaging workload so that those who are keen are able to take up interventional radiology.

Partly because the workloads are still not sufficient, most departments in all sectors do not have separate units/divisions for interventional radiology. Additionally, interventional radiology is still very young, with the constant turnover and the need to be still a competent general radiologist for private practice; most do not wish to take the plunge. The interventional radiologists still spend a significant proportion of their time doing diagnostic work encompassing all the modalities. More importantly in the private sector, the compensation structure still favours diagnostic studies instead of the interventional procedures. It is not uncommon for these interventional radiologists to perform their procedures after completing the diagnostic work. Due to the lack of numbers and, more importantly, expertise, there is no separation between the neuro-interventionalists and body interventionalists, and most do both.

Over the years, as the number of cardiologists has increased, so has their involvement in the peripheral vascular interventional work. Now, some perform aortic stent grafts and carotid interventions. In the recent past, the vascular surgeons have also started performing these procedures.

Following the recent Asian-Pacific Congress of Cardiovascular and Interventional Radiology (APCCVIR), the Malaysian Society of Interventional Radiology was formed. The current President is Dr. Alex Tang. This society will hopefully further develop the number of interventional radiologists.

## TRAINING AND CREDENTIALING

Under the Medical Act 1971, all medical practitioners who practise in Malaysia must be registered with the Malaysian Medical Council. This register has defined criteria for registration which is based on their basic medical degrees, whether specialists or non-specialists. The Act, as it exists, has no provision for a specialist register.

For specialists working in its hospitals and healthcare facilities of the MOH, the Government has its own gazettement exercise which is a requirement under the General Order under the Malaysian Public Services commission. This system has been operating well for the MOH. Unfortunately, this requirement does not apply legally to non-MOH organisations and the private sector. Different institutions may have individual processes and criteria and, therefore, differing standards. The universities have a similar but yet separate system where the criteria for appointment of specialists follow closely those of MOH. There is a special board under the management of each university, which has the power to approve confirmation as specialist and payment of specialist allowances. As for the private sector, there are no legal requirements beyond registering as a qualified practitioner. Each institution has its own privileging committee, which decides on the scope of practice of each specialist working in that institution. Thus, different institutions with their individual processes and criteria may result in differing standards.

The MOH along with the Malaysian Medical Council (MMC) and the Academy of Medicine Malaysia has put in place the processes and structure for a National Specialist Register [7]. Similar to those of Hong Kong and Singapore, this register has been implemented to ensure that doctors who are designated as specialists are appropriately trained, and fully competent to deliver the expected higher level of care in the chosen specialty.

Until the new Medical Act is passed, credentialing of specialists will be performed by the National Credentialing Committee (NCC), established in the MOH under the chairmanship of the Director General of Health, Malaysia. The NCC consists of members from both the MOH and Academy of Medicine of Malaysia. Upon passage of the new Medical Act, the MMC will ensure that those admitted to the National Specialist Register (NSR) are competent and fit to practice. Thus far, there were more than 44 specialty subcommittees as listed in the NSR website. Radiology has been registered as a speciality under the current structure with no provision for any subspecialty as the numbers are too small and the subspecialty practices are still in their infancy.

Training of interventional radiologists has also been fragmented with the different sectors following their own programme. The MOH has embarked on a 3-year programme of supervised clinical training in a local institution for 2 years and 9 months to a year abroad mostly in the UK or in Australia. Following this period of approved training, the MOH will then recognise them as subspecialists following additional requirements. Currently, there are three qualified interventional radiologists with four in training and an additional three who will be joining soon [8]. The MOH has been continuously losing these specialists to the private sector due to the more attractive remuneration packages.

The universities, thus far, have been sending their interventional radiologists for fellowship programmes. These informal programmes are also for 1–2 years with a period of between 1 and 2 years in the UK, Singapore or Australia. There is still no formal system of recognition as subspecialists within the universities and neither is there a promotion or increased compensation [9]. The practicing interventional radiologists in the private sector have generally trained while within the MOH or from one of the local universities prior to changing their practice. As mentioned previously, each institution formulates its own privileges as to the scope of interventional practice.

With regards to the private sector, each hospital has its own credentialing body which accords the interventional radiologist practicing privileges. There has been a gradual trend for these hospitals to seek interventional radiologists preferentially when looking for radiologists. However, these radiologists still do a significant amount of diagnostic work in their practices.

## FUTURE CHALLENGES AND PROSPECTS

The future of healthcare poses challenges, which include a changing disease pattern, a well-informed and demanding public, rising costs, new medical technologies, globalisation and liberalisation. Access to healthcare and their effectiveness to meet the needs of the population is largely dependent on how healthcare is organised and delivered, and what type of medical technology is used to make the delivery more efficient. On both these counts, a comprehensive and integrated interventional radiology service built on appropriate imaging technologies will be able to play a pivotal role in solving some of these challenges. The MOH, thus far, does not have a national policy on the development of interventional radiology based on the country’s needs and, as such, the efforts have been piecemeal.

Today, there are pockets of interventional services being provided in different institutions at different time points but there is distinct lack of a national framework for the development of the speciality. Even though careful planning and development efforts have enabled the country to progress through various phases of modernisation, in line with changing demographics, socio-economic and technological challenges, there is a lack of coherent national programme in the development of interventional radiology. This is a major limiting factor for the growth of interventional radiology. Thus, differences between the different stakeholders i.e. the MOH, universities and private institutions, have been allowed to perpetuate and have hampered the development of such a framework.

The universities have a “local” perspective of interventional radiology and are looking at meeting their needs. One of the major shortcomings limiting the progress, not just of interventional radiology, is the distinct lack of “political” influence with the decision makers. It is, therefore, difficult for growth of the specialty. Differences in the view-points and, more importantly, personalities within the interventional community are also issues. The radiological community has their hands full with issues of manpower, turf, certification and continuing professional development, etc., that issues of the interventional community (though recognised as being essential for the growth of the speciality) are difficult to move forward.

Full-time interventional radiology in the private sector is not sustainable unless there are specialised private centres e.g. neuro-intervention. Efforts are being made through the College of Radiology, Academy of Medicine of Malaysia, to change the compensation structure for interventional procedures to make the specialty more financially attractive to the younger practicing radiologist.

The current generation of practicing doctors and future radiologists belong to a different generation, the so-called Generation-Y [10]. These generational differences are already starting to have major implications on their education, training and practice of not just diagnostic radiology but certainly on interventional radiology. It is difficult to find the young radiologists who are keen to take up intervention as they value a balanced lifestyle with time for family. There is also a significant change in the gender balance in radiology, not just in medical schools.

In an effort to overcome some of these confusing and differing views with regard to subspecialty training for interventional radiology, the University of Malaya through the Department of Biomedical Imaging, Faculty of Medicine, has offered a 4-semester program called an Advanced Master in Radiology in all the different specialties including interventional radiology. This is a structured programme, which consists of course work, clinical training and a research component. The programme is scheduled to start in 2010.

In the background of increased accountability and cost-pressures, the introduction of newer treatment modalities, advent of lesser invasive treatment modalities e.g. FUS and an increasing number of other specialties interest in imaging guided intervention e.g. neuro, interventional radiology faces a tremendous pressure to hang on to its slim lead. There has been increasing involvement of cardiologists and vascular surgeons in performing peripheral vascular and aortic stenting. There have also been efforts by the neurosurgeons to want to perform neuro-intervention in major public hospitals. There is currently a halt to this.

Unless all the players in this drama can come together and convince the MOH of the importance of interventional radiology in improving outcome and at the same time reducing costs of treatments, the future of interventional radiology in Malaysia does not look promising but only time will tell.

## References

[1] Ministry of Health, Malaysia (2000). Health financing. Finance Division, Ministry of Health Malaysia.

[2] Ministry of Health, Malaysia (2002). Financing healthcare in Malaysia: The way forward.

[3] World Health Organization (2000). Health systems: Improving performance. World Health Report 2000. World Health Organization.

[4] Ministry of Health, Malaysia Fakta Kesihatan.

[5] National QA Program.

[6] Ministry of Health, Malaysia (2004). Indicators for monitoring and evaluation of strategy for health for all. Annual report.

[7] Malaysian National Specialist Register.

[8] Musa Z Personal communication. Radiological Services Ministry of Health, Malaysia, 28th August 2009..

[9] Ramli N Personal communication. Department of Biomedical Imaging, University of Malaya, Kuala Lumpur, Malaysia, 28th August 2009..

[10] Abdullah BJJ (2009). Generational challenges to radiology education and practice. Biomed Imaging Interv J.

